# Oral Microbiome as a Tool of Systemic Disease on Cleft Patients: A New Landscape

**DOI:** 10.7759/cureus.35444

**Published:** 2023-02-25

**Authors:** Vania Arboleda, Kawther N Elsouri, Samantha E Heiser, Isabel Bernal, Marc M Kesselman, Michelle Demory Beckler

**Affiliations:** 1 Osteopathic Medicine, Nova Southeastern University Dr. Kiran C. Patel College of Osteopathic Medicine, Fort Lauderdale, USA; 2 Osteopathic Medicine, William Carey University College of Osteopathic Medicine, Hattiesburg, USA; 3 Rheumatology, Nova Southeastern University Dr. Kiran C. Patel College of Osteopathic Medicine, Fort Lauderdale, USA; 4 Microbiology and Immunology, Nova Southeastern University Dr. Kiran C. Patel College of Allopathic Medicine, Fort Lauderdale, USA

**Keywords:** plastic and reconstructive surgery, respiratory tract infections, caries & periodontal disease, oral microbiome, cleft lip palate

## Abstract

The oral cavity microbiome comprises benign and pathogenic bacteria, with more than 700 species identified. However, the current literature regarding resident bacterial flora in the oropharyngeal cavities in cleft lip/palate (CLP) patients still needs to be completed. This review aims to evaluate the role of the oral microbiome of cleft patients as an indicator in systemic diseases for which cleft patients might be at higher risk in the short or long term.

A literature review was performed in July 2020 using Biomedical Reference Collection Comprehensive, Cumulative Index to Nursing and Allied Health Literature (CINAHL) Complete, Dentistry & Oral Sciences Source via Elton B. Stephens Company/Online Database (EBSCO), Turning Research into Practice (TRIP), and PubMed. The keywords used were "oral, bacteria, microbiome, biota, flora, cleft, palate." The resulting 466 articles were deduplicated using Endnote. The total amount of articles' abstracts without duplicates was filtered using a set criterion. The title and abstract filter criteria included 1) cleft lip (CL) and/or cleft palate (CP) patients, 2) changes in the oral microbiome in CL and/or CP patients, 3) male and female patients 0-21 years old, and 4) English language. The full-text filter criteria included 1) CL and/or CP patients vs. non-cleft control patients, 2) oral bacteria, 3) nonprocedural measurements of microorganisms, and 4) case-control studies. A Preferred Reporting Items for Systematic Review and Meta-Analyses (PRISMA) flow chart was created using the EndNote data results.

The final five articles of the systematic search indicated that the oral cavity of cleft lip and/or palate patients resulted in 1) contradicting levels of *Streptococcus mitis* and *Streptococcus salivarius*; 2) lower levels of *Streptococcus gordonii, Bordetella dentium, Fusobacterium nucleatum, Veillonella parvula, Bacillus *and *Lautropia* when compared to the control group; 3) higher levels of *Staphylococcus epidermidis* and Methicillin-sensitive *Staphylococcus aureus* compared to the control group; 4) presence of *Enterobacter cloacae* 36.6%, *Klebsiella pneumoni* 53.3%, and *Klebsiella oxytoca* 76.6% vs. absence in the control non-cleft group.

Patients with CL and/or CP are at higher risk for caries, periodontal diseases, and upper and lower respiratory infections. The results from this review indicate that relative levels of certain bacteria may be associated with these issues. The lower levels of *S. mitis, S. salivarius, S. gordini, *and *F. nucleatum* in the oral cavity of cleft patients could be linked as a possible cause of the higher incidence of tooth decay, gingivitis and periodontal disease as high levels of these bacteria are associated with oral disease. Further, the higher incidence of sinusitis in cleft patients might be linked to low levels of* S. salivarius* in the oral profile of these patients. Likewise, *E. cloacae, K. oxycota, *and *K. pneumoni* have been linked with pneumonia and bronchiolitis, both of which are increased in cleft patients. The oral bacterial dysbiosis of cleft patients observed in this review may play a vital function in the oral microbiome's diversity, which could play a role in disease progression and disease markers. The pattern seen in cleft patients potentially demonstrates how structural abnormalities can lead to the onset of severe infection.

## Introduction and background

Worldwide, oral clefts occur in about one in every 700 live births [1]. In the United States, approximately one in every 1,600 babies is born with cleft lip (CL) with or without cleft palate (CP) annually [2]. According to a recent article published by Fell et al. (2022), surgery to repair a CL occurred with a median age of 4.3 months [3]. Meanwhile, CP repair surgery is recommended within the first 6 to 12 months and tends to have a bimodal age distribution [3-4]. Surgical repair can improve the anatomy and physiology of a child's health. However, surgical repair is just the beginning of their comprehensive health.

Cleft children are at risk for bacterial infections that may cause severe complications. Post-surgical health care needs for cleft patients include but are not limited to breathing, hearing, and speech therapy [4]. Families with post-surgical cleft children might have to deal with early complications such as asphyxia, pyrexia, upper respiratory tract infections, bronchiolitis, pneumonia, hemorrhage, odontoptosis, or periodontitis [5]. Furthermore, long-term complications include secondary lip/nasal deformity, dehiscence of the lip, palatal fistula/decencies, hearing problems or otitis media, poor ventilation or snoring, velopharyngeal incompetence, and voice disorders [5-8]. These acute and chronic healthcare complications have serious consequences, resulting in short and long-term life quality and expectancies differences, which distress the patient and their family and present an extra socioeconomic burden [9].

The oral cavity of CL and/or CP patients is one of the primary culprits for many benign and pathogenic bacteria, as there are more than 700 bacteria species in this anatomical region [10]. The current literature remains incomplete regarding the impact of resident bacterial flora in the oropharyngeal cavities of cleft patients and its value as a possible indicator for systemic diseases [11,12]. The oral cavity of CL and/or CP patients is polymicrobial [13,14]. However, these patients' oral microbiome profiles can be valuable for identifying pathogenic and commensal bacteria as potential association with systemic healthcare complications. We hypothesized that dysbiosis of the oral microbiome of cleft patients might indicate systemic diseases for which cleft patients might be at higher risk in the short or long term.

## Review

Methods

**Figure 1:**
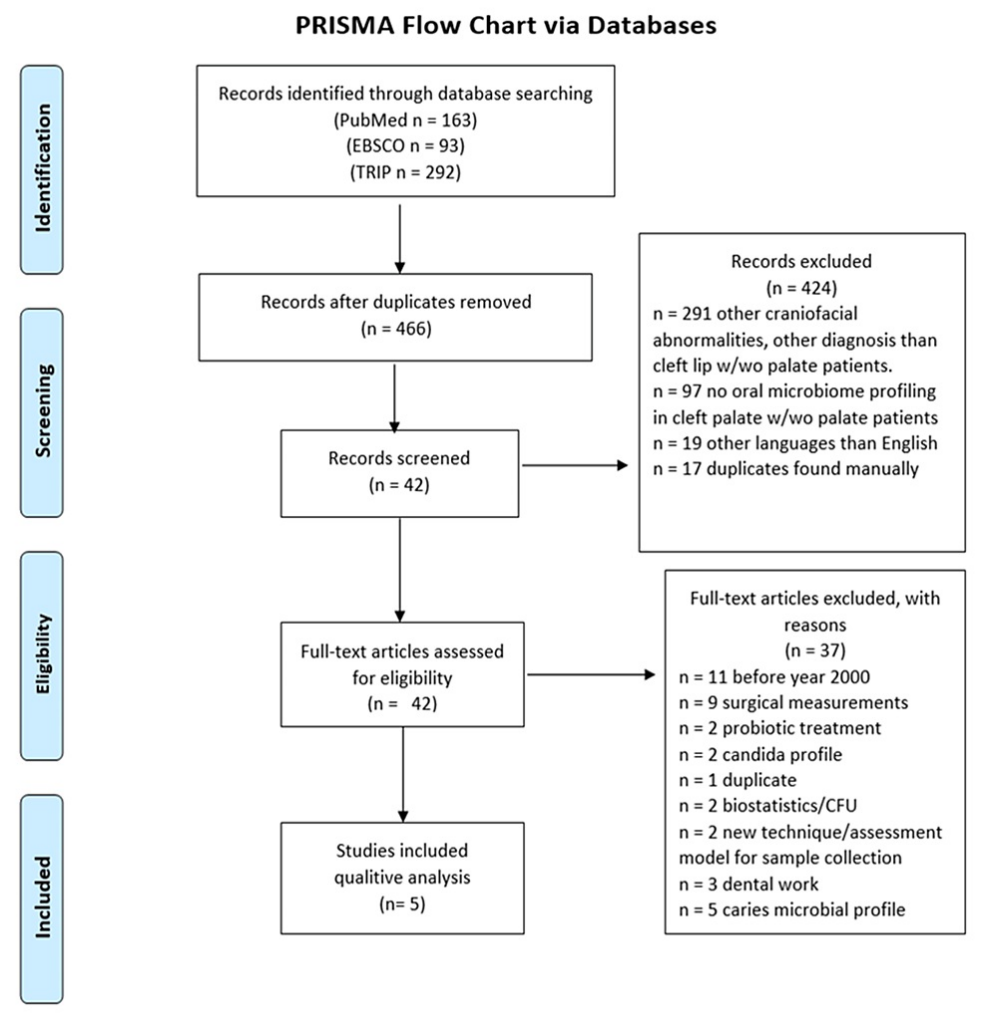
PRISMA Flow Chart via Databases: Biomedical Reference Collection Comprehensive, CINAHL Complete, Dentistry & Oral Sciences Source via EBSCO, TRIP, and PubMed. PRISMA: Preferred Reporting Items for Systematic Review and Meta-Analyses. CINAHL: Cumulative Index to Nursing and Allied Health Literature. EBSCO: Elton B. Stephens Company/Online Database. TRIP: Turning Research into Practice.

A literature review was performed in July 2020 using Biomedical Reference Collection Comprehensive, Collection Comprehensive, Cumulative Index to Nursing and Allied Health Literature (CINAHL) Complete, Dentistry & Oral Sciences Source via Elton B. Stephens Company/Online Database (EBSCO), Turning Research into Practice (TRIP), and PubMed. The keywords used were “oral, bacteria, microbiome, biota, flora, cleft, palate.” The resulting 466 articles were deduplicated using EndNote. The total amount of articles’ abstracts without duplicates was filtered using a set criterion. The title and abstract filter criteria included 1) CL and/or CP patients, 2) changes in the oral microbiome in CL and/or CP patients, 3) male and female patients 0-21 years old, and 4) English language. If the articles’ abstracts focus on 1) other congenital anatomical abnormalities than CL and/or CP, 2) changes in the gut microbiome, 3) male and female patients older than 21 years old, 4) other languages than English, 5) non-human oral microbiome changes, the articles were excluded. The full-text filter criteria included 1) CL and/or CP patients vs. non-cleft control patients, 2) oral bacteria, 3) nonprocedural measurements of microorganisms, and 4) case-control studies. Articles were excluded if they were 1) case series/reports, review articles, clinical trials, retrospective, or other studies; 2) yeast, virus, fungi, or other microorganisms than bacteria measurements; 3) procedural measurements of bacteria, caries, surgeries, procedures; 4) no statistical analyses; 5) antibiotic, experimental, other treatments influence oral bacteria; 6) articles before 2000. A Preferred Reporting Items for Systematic Review and Meta-Analyses (PRISMA) flow chart was created using the EndNote data results (Figure 1).

Results

The systematic search (Figure 1) resulted in five case-control studies comparing the oral microbiome in diverse types of cleft patients vs. non-cleft controls aged 0-21 years old (Table 1). These five case-control studies focused on contrasting and comparing the oral microbiome between cleft patients and the non-cleft controls. In all studies, neither groups underwent procedures nor pharmaceutical treatment during the period for which the five studies performed analysis.

**Table 1:** Summary of systematic search of final five articles. Source: References [15-19] CLP: cleft lip palate. UCLP: unilateral cleft lip palate. BCLP: bilateral cleft lip palate. CP: cleft palate. CL: cleft lip. CSP: cleft soft palate. yo: years old. mo: months. *P. nigrescens: Prevotella. S. mitis: Streptococcus. S. salivarius: Streptococcus. S. aureus: Staphylococcus. *MSSA: Methicillin-Sensitive* Staphylococcus aureus. S. epidermidis: Staphylococcus. E. cloacae: Enterobacter. K. pneumoni: Klebsiella. K. oxycota: Klebsiella. S. gordonii: Streptococcus. B. dentium: Bordetella. F. nucleatum: Fusobacterium. V. parvula: Veilonella. *

Year	Title/Author	Design	Cleft	Control	Age Cleft	Age Control	Country	Cleft Type	Samples	Results
2003 [15]	Clinical and microbiological evaluation of the periodontal status of children with unilateral complete lip and palate. Costa et. al.	Case-Control	30	27	5-6 yo	5-6 yo	Brazil	30 unilateral CLP	subgingival	No statistical sig. changes in *P. nigrescens* between groups (p = .709)
2009 [16]	Oral health status and behavior of Greek patients with cleft lip and palate. Parapanisiou et. al.	Case-Control	41	41	6-18 yo	6-18 yo	Greece	5 isolated CP, 26 UCLP, 10 BCLP	saliva	No statistically sig. changes in *Streptococcus mutants* & Lactobacillus levels between groups.
2016 [17]	Profiling of oral and nasal microbiome in children with cleft palate. Zhang et. al.	Case-Control	10	10	1-2 yo	1-2 yo	China	10 complete CP w/ or w/out CL	saliva	Statistically sig. changes in *Bacillus* and *Lautropia*, between cases vs controls.
2017 [18]	A comparative study of oral microbiota in infants with complete cleft lip and palate or cleft soft palate. Machorowska et. al.	Case-Control	30	25	0 yo - 4-90 days	4-90 days	Poland	30 CLP, 25 CSP	gingival	Statistically sig. changes in *S. mitis, S. salivarius, S. aureus *MSSA*, S. epidermidis, E. cloacae, K. pneumoni, K. oxytoca* between cases vs controls
2018 [19]	Salivary microbial profiles in 5-year-old children with oral clefts: a comparative study. Sundell et. al.	Case-Control	80	144	5 yo	5 yo	Sweden	12 isolated CP, 68 CP w/ or w//out CL	gingival	Statistically sig. changes in *S. mitis, S. gordonii, S. salivarius, B. dentium, F. nucleatum, V. parvula* between cases vs controls

Costa et al. [15] evaluated cleft patients (N=30) and non-cleft patients (N=27) for three species of bacteria via standard antigens: *Porphyromonas gingivalis, Prevotella nigrescens, *and *Treponema denticola. *The median age of participants was five and a half years old (Table 1). *P. gingivalis *and *T. denticola* were not detected on the sub-gingival samples of either cleft or non-cleft controls. Five of 30 children in the experimental group (16.67%) and three of 27 children in the control group (11.11%) tested positive for *P. nigrescensin.* Costa et al. found no significant level changes in *P. nigrescens* between control and cleft patients (Table 1).

Parapanisiou [16] evaluated cleft patients (N=41) and non-cleft patients (N=41) for two bacteria via stimulated saliva samples: *Lactobacilli *and *Streptococci mutants.* The median age of participants was 12 years old (Table 1). They found no significant level changes in *Streptococcus mutants*nor *Lactobacillus *between cleft versus control groups (Table 1).

**Figure 2:**
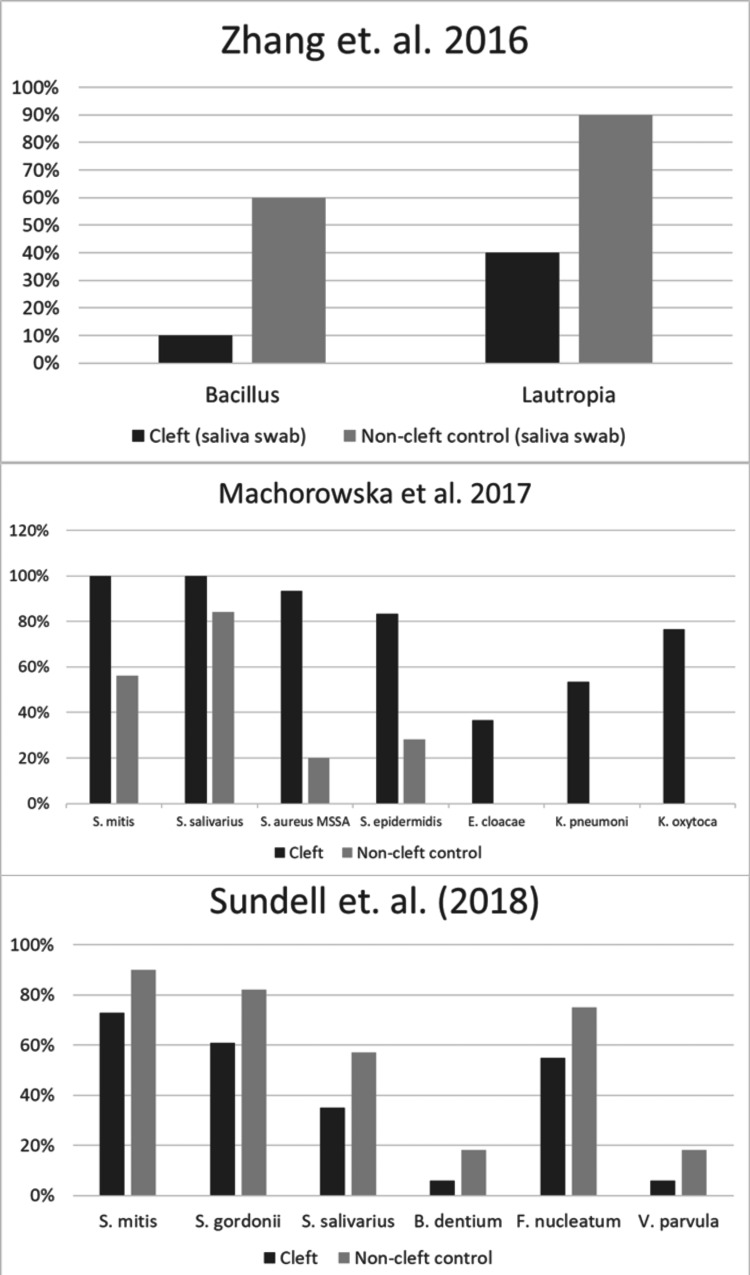
Oral bacterial profile differences between cleft patients vs. control group organized by year and the article in which the data was published *S. mitis: Streptococcus. S. salivarius: Streptococcus. S. aureus: Staphylococcus. *MSSA: Methicillin-Sensitive *Staphylococcus Aureus. S. epidermidis: Staphylococcus. E. cloacae: Enterobacter. K. pneumoni: Klebsiella. K. oxycota: Klebsiella. S. gordonii: Streptococcus. B. dentium: Bordetella. F. nucleatum: Fusobacterium. V. parvula: Veilonella. * Zhang et al [17], Machorowska-Pieniążek et al. [18], Sundell et al. [19]

Zhang et al. [17] performed a total microbial genomic DNA profile of the saliva of cleft (N=10) and non-cleft patients (N=10). The median age of participants was 22 months old (Table 1). They were able to distinguish 10 bacteria genera: *Dolosinogranulum, Streptococcus, Moraxella, Gemelli, Staphylococcus, Neisseria, Corynebacterium, Rothia, Lautropia, *and *Bacillus. *They only found statistically significant level changes of *Bacillus *(cleft 10% vs. non-cleft 60%) and *Lautropia *(cleft 40% vs. non-cleft 90%) between the cleft vs non-cleft control group (Figure 2).

Additionally, Machorowska-Pieniążek et al. [18] performed a comprehensive bacteria profile using gingival samples of cleft (N=30) and non-cleft patients (N=25). The median age of participants was 47 days old (Table 1). Machorowska-Pieniążek et al. distinguished 13 bacteria genera: *Streptococcus, Lactobacilli, Gemella, Enterococcus, Staphylococcus, Neisseria, Moraxella, Acinetobacter, Enterobacter, Serratia, Klebsiella, Citrobacter, *and *Escherichia.* Machorowska-Pieniążek et al. found significantly higher levels of *S. mitis *(cleft 100% vs. 56% non-cleft), *S. salivarius* (cleft 100% vs. non-cleft 84%), and *S. epidermidis* (cleft 83.30% vs. non-cleft 28%), MSSA (cleft 93.30% vs. non-cleft 20%), *E. cloacae* (cleft 36.6% vs. non-cleft 0%), *K. pneumoni* (cleft 53.3% vs. non-cleft 0%), and *K. oxytoca* (cleft 76.6% vs. non-cleft) (Figure 2).

Lastly, Sundell et al. [19] evaluated whole genomic DNA probes of cleft (N=80) and non-cleft (N=144) participants prepared from a panel of seven bacteria genera: *Actinomyces, Bifidobacterium, Fusobacterium, Lactobacilli, Rothia, Streptococcus, *and *Veilonella.* The median age of participants was 5 years old (Figure 2). They found significantly lower levels of *S. mitis* (cleft 73% vs. 90% non-cleft), *S. gordonii *(cleft 61% vs. non-cleft 82%), *S. salivarius* (cleft 35% vs. 57% non-cleft), *B dentium* (cleft 6% vs. 18% non-cleft), *F. nucleatum* (cleft 55% vs. 75% non-cleft), and *V. parvula* (cleft 6% vs. 18%) when compared to their non-cleft control (Figure 2).

Together, these results show significantly different levels of bacteria in the oral cavity compared to their control group. The profile from this systematic search and review resulted in the oral cavities of CP with or without CL patients having lower levels of the* Streptococci: S. mitis, S. salivarious, S. gordini, Bacillus, Lautropia, Bordetella dentium, Fusobacterium nucleatum, *and *Veillonella parvula*, while having higher levels of the *Staphylococci - S. epidermitis *and MSSA* -* when compared to non-cleft patients. Furthermore, patients with CP with or without CL had the presence of *Enterobacter cloacae, Klebsiella pneumoni, *and *Klebsiella oxycota* versus the complete absence of these pathogens when compared with the control group.

Discussion

In the search for the profile of CL with or without CP patients, five articles compared and contrasted oral microbiome samples from cleft patients vs. non-cleft patients. Neither group underwent treatment during the period for which the five studies performed analysis. However, a limitation of our results lies in the microbial profiles of the articles included. While Zhang, Machorowska-Pieniążek et al., and Sundell [17-19] profiled whole genomes of the oral microbiome of participants, Costa and Parapanisiou [15-16] measured findings for specific bacteria. While our study searched and filtered through all articles related to this topic, we cannot objectively compare the findings of studies with such a difference in methodology. It is due to this reason that we can only discuss and compare the findings of studies done by Zhang, Machorowska, and Sundell. Indeed, these case-control studies provide a baseline that while the oral cavity of CL with or without CP patients is polymicrobial, cleft patients have some significant differences when contrasted with non-cleft patients. These differences might be associated with caries, periodontal disease, and upper and lower respiratory infections.

Caries and Periodontal Disease

Dental caries is the medical term for tooth decay resulting from tooth-adherent cariogenic bacteria [20]. Excessive acidification of the oral environment by aciduric species such as *Streptococcus mutans *is directly associated with the development of dental caries [21]. Oral *Streptococci* are early colonizers of the oral cavity with low pathogenic potential. This group of bacteria can metabolize carbohydrates via fermentation, generating acids as byproducts [10]. *Streptococcus mitis'* main goal is to break down excess carbohydrates to prevent the over-acidification of the oral cavity. Further support is provided by *Streptococcus salivarius* and S*treptococcus gordonii*. These two bacteria produce large amounts of alkali, displaying an essential role in the acid-base physiology of the oral cavity [10]. Imbalances in the oral *Streptococci* in CL and/or CP patients present a risk factor for caries in CL and/or CP patients [22-25]. Cleft patients have as high as twice the risk for caries as their non-cleft counterparts [26,27]. This higher incidence presents a long-term physical and socioeconomic burden that affects the patient's oral health and places economic stress on the patient's parents. Although we have highlighted the significance of *S. salivarious *and *S. gordini *and their protective role in caries, further research should focus on their role in the higher-risk CL and/or CP patients toward tooth decay. 

There was a discrepancy in the levels of *Streptococcus mitis *and *Streptococcus salivarius *in the profile of patients with CL with or without CP. Machorowska-Pieniążek et al. et al. published higher levels of both pathogens, while Sundell et al. published lower levels of the same pathogens. Although both groups studied gingival samples [28], some of the differences between them were the cleft type and the age of the subjects studied. Machorowska-Pieniążek et al. studied zero to 90 days old CP with CL patients, while Sundell et al. studied five years old CP with or without CL patients. These age profile differences might indicate that newborns with CL with CP patients are born with higher protective levels of *Streptococci* which diminish with aging. *S. salivarius* is one of the first commensal bacteria to establish itself in the oral cavity of a newborn [29]. These non-pathogenic bacteria tend to prevent the over-colonization of *Streptococcus mutans *and *Streptococcus sobrinus*, some of the virulent *Streptococci* responsible for tooth decay [29]. Low levels of *S. salivarious* might be associated with an earlier age risk of tooth decay in this population.

An unexpected finding was the lower levels of *Bordetella dentium* found on CP with or without CL. Although, the presence of *B. dentium* is a known contributor to extensive tooth demineralization by fermenting carbohydrates which can then acidify the oral pH, leading to the formation of caries [30]. This research highlights how oral cavity bacteria interact with one another to either prevent or halt the onset of disease conditions, where one organism benefits from or requires the other to survive. Thus, the balance of commensal and pathogenic bacteria plays a pivotal role in the maintenance of the oral cavity in cleft patients [11,31,32].

Per the Centers for Disease and Control Prevention, periodontal diseases are conditions that involve infections, attachment loss, and inflammation of the gingiva, along with other structures that surround the tooth [33]. Oral *Streptococci *produce an arsenal of adhesive molecules that allow these bacteria to efficiently colonize different tissues in the mouth including the gingiva [10]. These bacteria adherences act as a trigger for bacterial extracellular polysaccharides and extracellular DNA to build up the plaque biofilm matrix [10]. The biofilm formation in the oral cavity serves as an important barrier for pathogenic and opportunistic bacteria [34]. The lack of biofilm creates the perfect niche for bacteria to grow in and around the teeth area and surrounding areas. *S. gordinii *also plays an important role in oral biofilm formation [35]. *S. gordinii *possesses many adhesive structures, some key ones in their role in biofilm formation are GspB and Hsa, which are serine-rich repeated (Srr) glycoproteins that bind salivary mucin [10]. It is important to highlight that Srr proteins are not present in caries pathogens such as *S. mutans* [10], which showcases the importance of maintaining the protective role of *S. salivarius *at higher oral levels. This research also found low levels of a late colonizer *F. nucleatum *[36], which is known for its bridging role with *S. gordinii* in biofilm formation of healthy and diseased teeth and gingiva [37]. Lima et al. proposed that besides the RadD adhesin, the interaction between *F. nucleatum *and *S. gordinii *and their role in biofilm formation involves a second outer membrane protein, CmpA. CmpA acts as a dual-species biofilm formation with *S. gordinii *V288 to further form and maintain the biofilm matrix [37]. This symbiotic relationship might be at play when low levels of these bacteria are present in cleft patients. The balance between these bacteria in the oral cavity of CL with or without CP might play a direct role in the development of caries. Plus, the diminished biofilm capacity due to low levels of these bacteria may play a role in the higher incidence of gingivitis and periodontal diseases in CL and/or CP patients.

Children and adolescents with CL and/or CP have an increased prevalence of caries, gingivitis, and mild periodontitis [38]. Passinato Gheller et al. [38] agree that there is an increased prevalence of gingivitis and mild periodontitis in children and adolescents with CL and/or CP. The study further evaluates the plaque index, gingival bleeding index, probing pocket depths, and clinical attachment level, which indicate overall oral hygiene. The CL and/or CP group had significantly higher levels and incidences in each category compared to the control group. These findings support our associations, and it may suggest that the presence of CL and/or CP patients and the corresponding oral dysbiosis that comes with the anatomical changes might lead to an increase in tooth decay and gingival inflammation, potentially leading to an increase in caries, gingivitis, and periodontal diseases.

Upper Respiratory Diseases: Sinusitis

Sinusitis has been associated with cleft lip and palate [39-41]. Wei et al. showed maxillary sinusitis increases with age in cleft children, with the incidence of more severe sinusitis seen in children under 18 years old [41]. Further, Demirtas et al. published that children with CLP have a significantly thicker Schneiderian membrane and sinus mucosal thickness when compared to control groups [40]. Multiple theories support the increased prevalence of maxillary sinusitis in developing children [39,40]. Most acceptable, children with CL and/or CP may be born with a small maxilla which may cause malpositioning or narrowing of sinuses, potentially leading to sinusitis [42]. However, we propose the higher incidence may be related to oral microbiome dysbiosis, specifically, lower levels of* S. salivarius*. Strains of this bacterium have an anti-*Streptococcus* pyogenes activity role and have increasingly been used as preventative measures for sinusitis and upper respiratory tract infections such as tonsillitis and pharyngitis [43-45]. *S. salivarius *TOV-R strain's inhibitory activity towards *Streptococcus pyogenes and Streptococcus pneumoniae* has been directly linked to its bacteriocin production [29]. Additionally, *S. salivarius *affects immunity by inhibiting inflammatory pathways activated by these bacteria via inhibition of IL-8 secretion and innate immune response pathways in alveolar, bronchial and pharyngeal epithelial cells [29]. The diminished levels of* S. salivarius* might be an opportunity to provide probiotics to boost the immune protective role of *S. salivarius* against both upper and lower respiratory infections and decrease the higher incidence of sinusitis in CL and/or CP patients. 

Lower Respiratory Diseases: Bronchiolitis and Pneumonia

Lower respiratory tract infections (LRTI) are prevalent in children with CL and/or CP. Sato et al. found that children under one year of age with CL and CP have a higher incidence of pneumonia and bronchiolitis than those in the non-cleft control group [46]. *K. pneumoni *is an etiology of community-acquired pneumonia and hospital-acquired pneumonia [47]. The polysaccharide capsule of the organism is a crucial virulence factor as it allows the bacteria to evade phagocytosis and serum killing by the host [48]. *K. pneumoni *has historically been identified as a pathogen in hospital-acquired infections [48]. Higher levels of *K. pneumoni *were found in this research as part of the baseline oral profile in CP and/or CL patients, linking the higher incidence of pneumonia in this patient population.

Furthermore, higher levels of *E. cloacae *levels were found in CLP compared to the complete absence of the bacteria in the non-cleft controls.* E. cloacae* has been reported as an opportunistic and multi-resistant bacterial pathogen for humans in hospital wards for the last 30 years [49]. *Enterobacter *is a versatile bacterium that responds promptly to antibiotic treatment in the colonized patient when treated timely [49]. Mainly due to its ability to form biofilms and secrete various cytotoxins (enterotoxins, hemolysins, and pore-forming toxins) which is essential for its pathogenicity [50]. This bacterial species can acquire numerous genetic mobile elements that strongly contribute to antibiotic resistance [49]. Furthermore, *K. oxytoca *has recently emerged as a bacterial isolate causing hospital-acquired infection in adults with multiple drug resistance to commonly used antibiotics [51]. *K. oxytoca *has been isolated from different clinical samples, mainly from the blood and respiratory secretions, and is gaining clinical significance in immunocompromised and debilitated patients admitted to Intensive Critical Care Units (ICUs) [51]. The higher levels of *E. cloacae *and *K. oxycota *found in CLP patients and their high antibiotic resistance features could be related to the higher LRTI experienced by cleft patients [42]. This knowledge might be helpful for future clinical management of these patients to decrease the continued burden of higher incidence of LRTI. Further, targeting these bacteria on time could help decrease the frequency of complications and overall episodes experienced per year in this population.

## Conclusions

The oral cavity of CL and/or CP patients is polymicrobial. Oral bacteria dysbiosis plays a vital function in the oral microbiome's diversity, which could play a role in disease progression and disease markers. Cleft patients have some significant differences when contrasted with non-cleft patients. These differences in the oral microbiome of CL and/or CP might be associated with a higher incidence of caries, periodontal disease, and upper and lower respiratory infections.

The pattern seen in cleft patients potentially demonstrates how structural abnormalities can lead to the onset of severe infection. CL and/or CP patients and the corresponding oral dysbiosis that comes with the anatomical changes might increase the risk for caries, periodontal diseases, sinusitis, bronchiolitis, and pneumonia. Thus, it is vital to maintain a healthy balance between the commensal and pathogenic bacteria in the oral cavity of cleft patients to diminish their risk for these diseases.

Furthermore, this review highlighted three scenarios further research should focus on to reduce the short and long-term burden caries, periodontal, and upper and lower respiratory infections have on CL and/or CP patients: (1) To investigate the immune protective role of *S. salivarius *against upper and lower respiratory infections in CL and/or CP patients; (2) To analyze the protective commensal bacteria* S. salivarious *and *S. gordini'*s* *protective role in caries development in CL and/or CP patients; (3) To examine if on-time targeting of the resistant* E. cloacae *and *K. oxycota *decreases the higher incidence of LRTI in CL and/or CP patients.
